# Persufflation (gaseous oxygen perfusion) as a method of heart preservation

**DOI:** 10.1186/1749-8090-8-105

**Published:** 2013-04-22

**Authors:** Thomas M Suszynski, Michael D Rizzari, William E Scott, Peter M Eckman, James D Fonger, Ranjit John, Nicolas Chronos, Linda A Tempelman, David ER Sutherland, Klearchos K Papas

**Affiliations:** 1 Division of Transplantation, Department of Surgery, University of Minnesota, Minneapolis, MN, USA; 2 Department of Surgery, University of Texas Southwestern Medical Center, Dallas, TX, USA; 3 Institute for Cellular Transplantation, Department of Surgery, University of Arizona, 1656 E. Mabel Street, Room 121, Tucson, AZ 85724, USA; 4 Division of Cardiovascular Medicine, Department of Medicine, University of Minnesota, Minneapolis, MN, USA; 5 St. Josephs Translational Research Institute, Atlanta, GA, USA; 6 Division of Cardiothoracic Surgery, Department of Surgery, University of Minnesota, Minneapolis, MN, USA; 7 Giner Inc, Newton, MA, USA

**Keywords:** Organ preservation, Heart transplantation, Ischemia, Perfusion

## Abstract

Persufflation (PSF; gaseous oxygen perfusion) is an organ preservation technique with a potential for use in donor heart preservation. Improved heart preservation with PSF may improve outcomes by maintaining cardiac tissue quality in the setting of longer cold ischemia times and possibly increasing the number of donor hearts available for allotransplant. Published data suggests that PSF is able to extend the cold storage times for porcine hearts up to 14 hours without compromising viability and function, and has been shown to resuscitate porcine hearts following donation after cardiac death. This review summarizes key published work on heart PSF, including prospective implications and future directions for PSF in heart transplantation. We emphasize the potential impact of extending preservation times and expanding donor selection criteria in heart allotransplant. Additionally, the key issues that need to be addressed before PSF were to become a widely utilized preservation strategy prior to clinical heart transplantation are summarized and discussed.

## Introduction

Recent advances in organ preservation have enabled improved donor organ quality even in the setting of prolonged cold ischemia time (CIT). For example, strong evidence now exists and supports the use of hypothermic machine perfusion (HMP) during kidney preservation [1-5], and excellent results were recently attained in the first human clinical trials using HMP during liver preservation [6]. It is believed that the use of HMP can extend allowable periods of CIT without reducing the overall metabolic quality of the organ [5]. In the case of kidney transplantation, the extended time window may further simplify the logistics of organ transportation from the procurement site to the transplant center, and facilitate distribution of kidneys across United Network of Organ Sharing (UNOS) regions. In the case of heart transplantation (unlike kidney transplantation), extended CIT would enable transplantation of many more hearts since prolonged preservation times are detrimental for heart transplant as historical data suggest that poorer outcomes follow >4-6 hours of CIT [7]. The short allowable CIT poses significant logistical challenges and constrains heart allocation to a relatively small geographic area compared to other organs. Delays often occur at the procurement site following thoracotomy in lieu of communication with the transplant center to allow for preparation of the recipient for transplant, particularly in the case of a difficult reoperative chest. Furthermore, the current time constraints effectively preclude human leukocyte antigen (HLA) matching, which may offer significant advantages for long-term graft and patient survival [8]. The inability to extend cold storage time may also limit successful organ placement. With prospective heart transplant recipients filling waiting lists, some of whom are not eligible for ventricular assist devices, the need to increase the number of transplantable hearts is pressing.

A key area of research lies in the optimization of oxygen delivery during hypothermic preservation. It has been shown that conventional static cold storage (SCS) techniques are incapable of providing sufficient oxygen to the core of a large (human-sized) organ, and can only oxygenate to a maximum approximate depth of a millimeter from the surface [9]. Most efforts to improve the oxygen solubility of cold preservation solutions by using perfluorocarbons have proven largely ineffective, because these methods still rely on oxygen delivery by passive diffusion from the surface alone. Even HMP, which has been designed to deliver cold preservation solutions into the organ via the native vasculature, may fail at delivering adequate oxygen to an organ during preservation, such as when the perfusate is not saturated with oxygen at a higher than atmospheric oxygen partial pressure [10]. It is in this regard that persufflation (PSF) or gaseous oxygen perfusion may provide additional advantages as compared to either SCS or HMP. Although not a new concept, PSF can be considered an emerging (or reemerging) technique for organ preservation and deserves considerable attention for a variety of compelling reasons, including the unique capability to deliver oxygen gas or gas mixture directly into an organ by using the native vasculature. *Compared with SCS and HMP, PSF may represent the best opportunity to fully oxygenate a human-sized organ, such as the human donor heart prior to clinical transplant*.

**Figure 1:**
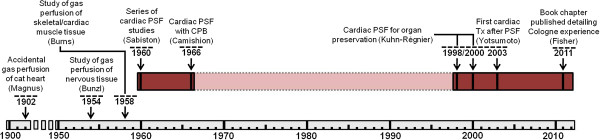
**Historical timeline of persufflation.** Significant contributions to the development of persufflation as a method of heart preservation.

The earliest experimental uses of oxygen PSF involved the heart, particularly as a preservation technique during experimental cardiopulmonary bypass (CPB). These initial studies demonstrated that gaseous oxygen can be utilized by myocardial tissue during periods of ischemia and that heart function can be restored following short-term PSF. Between the late 1960s and 1990s, investigation of PSF for heart preservation had slowed. The reasons for this are somewhat unclear, but perhaps the utility of PSF with other organs (i.e., kidney, liver) garnered more appeal during this interim. Recently, however, cardiac PSF has experienced rekindled interest. As detailed in this review, several studies have been published recently in which PSF was used in preservation prior to experimental heart transplantation. Among the more intriguing applications has been the use of PSF to preserve hearts following donation after cardiac death (DCD) and short periods of warm ischemia. A timeline of the more significant work in cardiac PSF is shown in Figure 1. The collective results over the years have, at minimum, suggested that cardiac PSF is technically feasible and has potential for preservation of heart tissue.

## Review

### Early history of persufflation

PSF was accidently discovered in 1902 by Rudolf Magnus while studying *ex vivo* blood perfusion of isolated feline hearts [11]. In an experiment, a blood-containing reservoir had unexpectedly emptied and compressed oxygen gas, which was used to drive blood perfusion, was pulled into the perfusion circuit and in through the coronary vasculature. Magnus observed that the heart continued to contract for several minutes while being perfused (or persufflated) with oxygen gas instead of blood.

These earliest observations gave way to a series of additional experiments performed by Magnus [11]. Not until the mid-1950s was PSF studied more extensively by a group at McGill University in Montreal [12,13]. In 1954, Bunzl *et al.* compared the benefits of PSF versus liquid perfusion in a frog spinal reflex model, showing that peripheral nerve reflexes and muscle contractions could be preserved for up to 6–8 hours with PSF [12]. At that time, the authors observed a lack of edema formation and improved oxygenation with PSF. In 1958, Burns *et al.* showed that rabbit heart function could be preserved for 3 hours using PSF [13]. This early work prompted others to pursue cardiac PSF.

### Early experiences in cardiac persufflation

In 1959, Sabiston *et al.* explored the use of PSF in conjunction with CPB, a technique that was new to cardiac surgery during that era [14]. In a first set of experiments, the authors showed that canine hearts would continue to beat for an average duration of 5 hours (2.5-8 hours, range) at 37°C using continuous *ex vivo* anterograde PSF (A-PSF) with humidified gaseous carbogen (95% O_2_, 5% CO_2_). They observed sustained cardiac contractility during the first 2–3 hours of PSF, with gradual weakening over time. In a second set of experiments, the authors performed *in situ* A-PSF for 25–30 minutes after which normal coronary blood flow was restored. They showed normal restoration of hemodynamic function in most animals, with several animals maintaining a heartbeat for 48 hours. *The key conclusions of this study were that 1) the heart is able use oxygen gas via PSF during periods of ischemia and 2) that normal hemodynamic parameters and coronary blood flow can be reestablished after PSF*.

**Figure 2:**
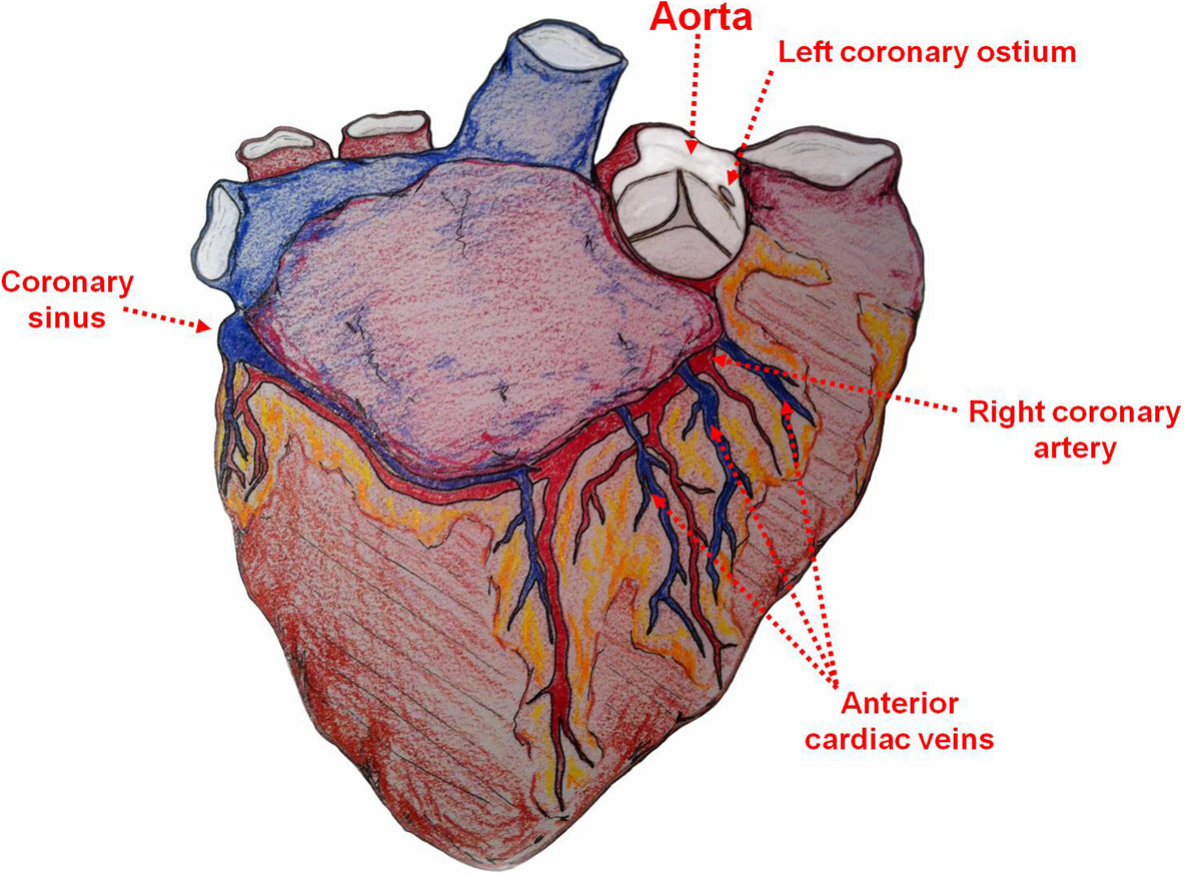
**Cardiac anatomy relevant to persufflation.** Sketch illustrating the cardiac anatomy that has been historically relevant to the development of persufflation; Anterograde persufflation has been performed via the aorta or direct cannulation of coronary ostia, whereas retrograde persufflation has been performed by cannulation of coronary sinus with or without cannulation of the anterior cardiac veins.

In 1960, Talbert *et al.* introduced the concept of retrograde PSF (R-PSF) [15]. At the time, retrograde perfusion of oxygenated blood via the coronary sinus was used to maintain a heartbeat and protect the heart from ischemia for short periods of time during open aortic valve procedures [16,17]. In their studies, R-PSF via the coronary sinus was able to maintain a visible heartbeat for an average duration of 3.5 hours (2–4 hours, range). If the anterior cardiac veins were cannulated in addition to the coronary sinus, R-PSF was even more effective; a visible beat was maintained for an average duration of 5.5 hours (Figure 2). The authors also compared R-PSF with A-PSF and determined that *the heartbeat was visibly weaker and sustained for a shorter period of time using the retrograde approach*, indicating that A-PSF may be the best-suited approach for heart preservation.

In 1966, Camishion *et al.* published an article that took Talberts’ concept of R-PSF and applied it during CPB [18]. Despite the earlier work displaying the superiority of A-PSF, there was a clear advantage to R-PSF within the context of CPB: R-PSF would not obstruct the surgical view over the aortic valve (because it did not involve cannulation of the coronary arteries). To address the question of whether R-PSF could preserve a heart during CPB, Talberts’ previous work on *in situ* R-PSF was basically repeated [15]. In a first experiment they found that R-PSF with oxygen gas maintained sinus rhythm in all canine hearts (n=10) for >30 minutes, and that R-PSF with nitrogen gas abolished sinus rhythm at ≤5 minutes. In a second experiment, 83% of canine or porcine hearts maintained sinus rhythm for one hour of R-PSF during CPB. Following removal of CPB, 73% remained in sinus rhythm. In addition to establishing the utility of R-PSF with CPB, *the authors cited no evidence of air embolization in the brain or viscera of any experimental animal. They also commented on the difference between gas embolization and free gas flow (which characterizes PSF),* one that is not yet fully appreciated by the clinical community and will need to be further validated by scientific investigation. Interestingly, the authors also speculated on the possible use of PSF for donor heart preservation prior to cardiac transplantation, nearly 2 years before the first successful human heart transplant [19]. Several other studies were conducted in the late 1960s, including Gabel *et al.* (1966) [20] and Lochner *et al.* (1968) [21] publishing on the metabolism and function of rat, feline and guinea pig hearts.

### Recent experiences in cardiac persufflation

During the next few decades (1970s-1990s), the use of PSF with kidney and liver became the focus of study in the field. The term ‘persufflation’ officially replaced ‘gaseous oxygen perfusion’ in 1971 [22]. Not until the late 1990s, however, was interest in cardiac PSF rekindled, which may have been a result of the successful application of PSF in other organs.

In 1998, Kuhn-Regnier *et al.* published a study exploring the use of A-PSF prior to orthotopic porcine heart allotransplantation [23], a related study to the one published by Fischer *et al.* that same year [24]. The mean preservation time in this study was 14.5 hours. They reported that persufflated hearts exhibited a cardiac stroke work capacity that was similar when comparing pre-transplant versus post-transplant (which was also post-PSF and post-CPB) function, whereas that was not the case in organs preserved by SCS due to severe arrhythmia and ventricular dyskinesia experienced post-transplant. Furthermore, cardiac output (CO) in persufflated hearts was significantly higher than in hearts preserved by SCS alone. Their data indicated that A-PSF permitted improved recovery of post-transplant cardiac function when compared with SCS. They also noted less myocardial edema with A-PSF versus SCS, which is important since tissue edema has been shown to significantly impair cardiac function [25]. In 2000, Kuhn-Regnier *et al.* performed another study in which they compared the metabolic quality and post-transplant function of hearts preserved by A-PSF versus SCS, and showed that both were significantly improved in transplanted hearts following A-PSF [26].

Up to this point in history, most studies of heart PSF involved experimental procedures with hearts experiencing no “down-time” or conventional warm ischemia as seen with DCD. The possibility of resuscitating DCD hearts as a way to make more donor hearts available for transplant became an intriguing concept. In 2003, Yotsumoto *et al.* studied the effect of 3 hours of A-PSF on post-transplant cardiac function following a mean warm ischemia time (WIT) of 16.7 minutes in a porcine (orthotopic) allotransplant model [27]. They discovered that SCS hearts following zero WIT and persufflated hearts following the prescribed WIT could be weaned from CPB within 2 hours of transplantation, whereas the SCS hearts following the prescribed WIT exhibited significantly worse function and could not be weaned. At 1-hour of reperfusion, troponin T levels were significantly higher in hearts preserved by SCS following the prescribed WIT, versus either SCS following zero WIT or A-PSF following the prescribed WIT. At the end of a 3-hour reperfusion period, all parameters of cardiac function (including CO, left ventricular contractility, and relaxation velocity) were significantly higher in hearts preserved by A-PSF as compared to SCS. Their studies indicated that A-PSF of a heart following DCD may enable preservation comparable to SCS of a heart following conventional donation after brain death (with no WIT).

A major concern of the transplant field has been the risk of endothelial damage imposed by PSF due to the introduction of pressured gas through the coronary vasculature. To address these concerns, studies were performed and have shown that the coronary arteries of porcine hearts following 3 hours of oxygen gas A-PSF exhibit normal endothelial function post-transplant [28-30]. Additionally, hearts transplanted following 14 hours of A-PSF showed no topographic signs of endothelial damage, as assessed by scanning electron microscopy [29]. Fischer recently published a book chapter in which the technical aspects of cardiac PSF are summarized [31] – including an important discussion regarding the development of an aortic valve guard to prevent gas efflux into the left ventricle during A-PSF via the coronaries. Table 1 summarizes the published work on heart PSF presented in this review.

**Table 1:** Summary of studies on heart persufflation

**Year**	**Author(s) [Ref.]**	**Approach for PSF**^**a**^	**WIT (min)**	**Duration of PSF (hours)**	**Model(s)**	**Transplant follow-up**	**Gas used**	**Temp (°C)**	**Primary endpoint**
*No transplant*									
1902	Magnus R [11]	A	-	≤ 1.15	Cat	-	O_2_, H_2_, CO_2_	24-28	Cardiac activity during PSF
1958	Burns *et al*. [13]	A	-	> 3	Rabbit	-	Carbogen^b^, N_2_	37	Cardiac activity during PSF
1959	Sabiston *et al*. [14]	A	-	< 8	Dog	-	Carbogen^b^, N_2_	37	Cardiac activity during PSF and after reperfusion
1960	Talbert *et al*. [15]	R	-	2-7	Dog	-	Carbogen^b^	37	Cardiac activity during PSF and reperfusion
1966	Camishion *et al*. [18]	R	-	< 7	Dog, Pig	-	O_2_, N_2_	38	Cardiac activity during PSF
1966	Gabel *et al*. [20]	A	-	10	Cat	-	Carbogen^b^	40	Cardiac activity and metabolic profile during PSF
1968	Lochner *et al*. [21]	A	-	< 1.5	Guinea pig, Rat	-	Carbogen^b^	4-37	Cardiac activity, WOOCR and metabolic profile during PSF
2001	Fischer J [28]	A	16	3.3	Pig (DCD)	-	O_2_	0-1	Coronary endothelial function
2004	Fischer J [30]	A	16	3.3	Pig (DCD)	-	O_2_	0-1	Coronary endothelial function
*Transplant studies*									
1998	Kuhn-Regnier *et al*. [23]	A	-	14.5	Pig	3 hours	O_2_	0-1	Cardiac function and metabolic profile post-allotransplant (orthotopic)
1998	Fischer *et al*. [24]	A	-	14.2	Pig	3-4 hours	O_2_	0-1	Cardiac function and metabolic profile post-allotransplant (orthotopic)
2000	Kuhn-Regnier *et al*. [26]	A	-	14.5	Pig	3 hours	O_2_	0-1	Cardiac function and metabolic profile post-allotransplant (orthotopic)
2003	Yotsumoto *et al*. [27]	A	16.7	2.3	Pig (DCD)	3 hours	O_2_	0-1	Cardiac function and metabolic profile post-allotransplant (orthotopic)
2004	Kuhn-Regnier *et al*. [29]	A	-	14	Pig	7 days	O_2_	0-1	Endothelial and myocardial cell function post-allotransplant (heterotopic)

*Superscripts*: ^a^Either anterograde via the coronary arteries (denoted by ‘A’) or retrograde via the coronary sinus and possibly anterior cardiac veins (denoted by ‘R’); ^b^Carbogen is defined as a gas mixture composed of 95% oxygen and 5% CO_2_. *Abbreviations*: DCD, donation after cardiac death; PSF, persufflation; WOOCR, whole organ oxygen consumption rate.

It is unclear why PSF has not been pursued with more vigor. It may be that a stigma accompanies the concept of introducing gas into the vasculature and, consequently, has made clinicians skeptical of the potential utility of PSF. There have been clinical trials in liver and kidney transplantation using PSF as a method of organ preservation, however these have been limited [32,33]. Perhaps concern for endothelial damage via desiccation is another limiting factor – though gases can be humidified and, as mentioned previously, many studies have shown that the endothelium does not appear disrupted and remains functional [28,30] even following long-term (14 hours) preservation by PSF and 7 days after heterotopic transplant [29]. Another possibility is that the logistics and safety concerns of transporting pressurized oxygen gas are major deterrents, especially during air travel, which is quite common and often necessary with donor heart allocation and transplant. However, recent technological advancements in developing portable devices that are able to generate *in situ* oxygen from water (via the use of electrochemical cells) have provided a method to obviate the issue of having to use pressurized gas cylinders. These devices have been used to generate breathable oxygen for occupants of deployed submarines and for patients with chronic pulmonary disease requiring home oxygen therapy, and have been modified for use in organ preservation (Giner Inc., Newton, MA) [34,35]. The devices are small, easily transportable and also safe to use in air travel. These technologies could enable PSF to become more widely used as a preservation method for organ transplantation. The increasing use of DCD organs for transplantation has unmasked a clear need for further advancement in organ preservation, an area of research that has been stagnant in recent years. This technology may prove to be a catalyst for such an advancement.

## Future implications and directions

Though the field of clinical transplantation has made considerable progress over the past half-century, an indisputable and persistent problem has been the donor organ shortage. Expansion of organ acceptance criteria, DCD, living donation, paired donor exchanges, and improving preservation methodology have all been strategies employed to increase the quantity of organs available for transplant [36-42]. Unfortunately, many of these strategies are not applicable in heart transplantation for obvious reasons, and suggest that improved organ preservation may be of particular importance in the case of the heart. It is conceivable that improved preservation techniques could lengthen the allowable CIT, which would provide some relief to the demanding logistical provisions that are needed during the allocation and transport of a donor heart. A substantial lengthening of the allowable CIT may even allow for HLA matching, which is currently not done routinely due to the limited time and may have a favorable impact on short- and long-term outcomes.

As described above, the opportunity to use PSF in the case of DCD hearts is intriguing. Currently it is perceived that the poor quality of the DCD heart does not merit the investment of resources required for their recovery and the added risk absorbed by the potential recipient. The opportunity to resuscitate organs damaged by prolonged WIT and to better prevent their deterioration during storage should provide the impetus to pursue the development of promising preservation strategies such as PSF. Even if improvements in preservation strategy do not lead to an immediate increase in the number of transplantable organs, an incremental improvement in this area should be welcomed. The motivation for this is reflected in the significant average attrition rate of the heart waitlist (due to deterioration in health or death) of about 20% per year (based on data from 2001–2009) (UNOS Data as of April 30^th^, 2010). Consequently, seeking better ways to recover and preserve a greater number of suitable organs should continue to be a primary objective.

To expand the acceptance and utilization of PSF in organ preservation, the technique must be further developed. Future work in PSF should involve: 1) continued optimization of technique and/or operational parameters (e.g., pO_2_, pressures, cannulation approach, etc.) so they are tailored to the tissue/organ-of-interest; 2) exploration of its use in conjunction with other preservation techniques (such as with HMP); or 3) as a method to condition organs prior to reperfusion (endischemic conditioning); 4) direct comparison with other well-accepted preservation techniques; 5) development of portable PSF systems (such as the electrochemical oxygen concentrator); 6) the identification of single or multiple pharmacologic agents used to prevent or reduce ischemia-reperfusion injury; and 7) confirmation that *ex vivo* PSF should not be considered analogous to *in vivo* gas embolization, and education of the clinical community regarding that embolization should not occur if PSF is performed properly.

In addition to its application with donor hearts prior to transplant, PSF may have utility during open cardiac surgery. As reviewed herein, the early works by Sabiston and Talbert *et al.*[14,15], and Camishion *et al.*[18] have inspired a reevaluation of the current method of preservation during cardiac standstill [31,43] and may suggest that PSF could improve outcomes following some or all cardiac surgery requiring CPB. The duration of cold ischemia involved during most coronary arterial bypass or valve procedures is relatively short (60–90 minutes) and does not seem to directly cause irreversible cardiac complications. Though supported by our current practice, this conventional understanding may need challenging. It could be that improved oxygenation during this relatively short period may reduce the complications that are believed to be associated with prolonged cross-clamp times (reduced ventricular compliance, poor inotropy, dysrhythmia, myocardial infarction, and others), but this hypothesis would need to be tested rigorously.

## Conclusions

As described in this review, oxygen gas delivered by PSF can be used by the heart during hypothermic preservation. PSF has shown that it may be capable of extending the allowable WIT and CIT and may maintain superior organ quality when compared with SCS. The basis behind the intervention of PSF is to provide an adequate oxygen supply to an organ during preservation. Data collected over decades has confirmed that improved oxygenation is better for maintaining the quality of an organ and, in some cases, enables the recovery of reversibly damaged tissue. Studies presented in this review have demonstrated PSF exhibits the capacity to improve or maintain the metabolic quality and function of the heart following procurement for transplantation. A major potential benefit to the use of PSF lies in the possibility to increase the number of suitable heart available for transplantation.

## Abbreviations

A-PSF: Anterograde persufflation; CIT: Cold ischemia time; CO: Cardiac output; CPB: Cardiopulmonary bypass; DCD: Donation after cardiac death; HMP: Hypothermic machine perfusion; HLA: Human leukocyte antigen; PSF: Persufflation; R-PSF: Retrograde persufflation; SCS: Static cold storage; UNOS: United Network for Organ Sharing; WIT: Warm ischemia time.

## Competing interests

TMS, MDR, WESIII, LT and KKP are co-inventors on published patent applications for technology associated with persufflation. LT is an employee of Giner, Inc. – which is a company that produces portable electrochemical oxygen concentrator units that can be used for persufflation. PME, JDF, RJ, NC and DERS have no conflicts of interest.

## Authors’ contributions

TMS, MDR, JDF, RJ, NC, LAT and KKP were involved in the conception of the review. TMS, MDR, WESIII, PME and KKP drafted the review. All authors were involved in revising the manuscript. TMS, PME and KKP provided final approval. All authors read and approved the final manuscript.
